# pH/Reduction Dual-Stimuli-Responsive Cross-Linked Micelles Based on Multi-Functional Amphiphilic Star Copolymer: Synthesis and Controlled Anti-Cancer Drug Release

**DOI:** 10.3390/polym12010082

**Published:** 2020-01-03

**Authors:** Yunwei Huang, Jingye Yan, Shiyuan Peng, Zilun Tang, Cuiying Tan, Jiabao Ling, Wenjing Lin, Xiaofeng Lin, Xihong Zu, Guobin Yi

**Affiliations:** 1 School of Chemical Engineering and Light Industry, Guangdong University of Technology, Guangzhou 510006, China; 2 Guangdong Provincial Key Lab of Green Chemical Product Technology, School of Chemistry and Chemical Engineering, South China University of Technology, Guangzhou 510640, China

**Keywords:** dual-responsive, cross-linked micelles, drug delivery, release kinetics, release thermodynamics

## Abstract

Novel approach has been constructed for preparing the amphiphilic star copolymer pH/reduction stimuli-responsive cross-linked micelles (SCMs) as a smart drug delivery system for the well-controlled anti-tumor drug doxorubicin (DOX) release. The SCMs had a low CMC value of 5.3 mg/L. The blank and DOX-loaded SCMs both had a spherical shape with sizes around 100–180 nm. In addition, the good stability and well pH/reduction-sensitivity of the SCMs were determined by dynamic light scattering (DLS) as well. The SCMs owned a low release of DOX in bloodstream and normal tissues while it had a fast release in tumor higher glutathione (GSH) concentration and/or lower pH value conditions, which demonstrates their pH/reduction dual-responsiveness. Furthermore, we conducted the thermodynamic analysis to study the interactions between the DOX and polymer micelles in the DOX release process. The values of the thermodynamic parameters at pH 7.4 and at pH 5.0 conditions indicated that the DOX release was endothermic and controlled mainly by the forces of an electrostatic interaction. At pH 5.0 with 10 mM GSH condition, electrostatic interaction, chemical bond, and hydrophobic interactions contributed together on DOX release. With the low cytotoxicity of blank SCMs and well cytotoxicity of DOX-loaded SCMs, the results indicated that the SCMs could form a smart cancer microenvironment-responsive drug delivery system. The release kinetic and thermodynamic analysis offer a theoretical foundation for the interaction between drug molecules and polymer matrices, which helps provide a roadmap for the oriented design and control of anti-cancer drug release for cancer therapy.

## 1. Introduction

Polymer micelles have shown their attractive advantages as anti-cancer nanocarriers for anti-cancer drugs and attracted more attention in recent years [1,2,3,4]. With the unique core-shell nanostructures, polymer micelles can enhance water solubility, raise drug-loaded capacity, reduce protein adsorption, prolong circulation duration, and increase drug accumulation at target sites and more [5,6,7,8,9]. However, there usually existed two weaknesses of traditional micellar systems. One is the premature drug release in bloodstream and normal tissues, and the other is the slow and incomplete drug release within cancer cells. These two weaknesses increase side-effects, shorten drug effective time, and reduce therapeutic efficacy.

To overcome these obstacles, a slate of stimuli-responsive cross-linked micelles (SCMs) self-assembled from amphiphilic copolymers were introduced. The SCMs with a cross-linked structure were more stable when compared with corresponding non-cross-linked micelles, which could prevent premature drug release in the blood vessel and normal tissues. When arriving at the target sites, the SCMs formed smart delivery systems and released drug rapidly by introducing stimulus-responsive groups into their polymers, which achieved high effective therapeutic effects [10,11,12,13,14,15,16]. In past years, with the advantages of stimulus-responsiveness, reversible chemical bonds, such as acetal, hydrazone, imine, diselenide bonds, disulfide, borate, and oxime bonds, are popular and have been used for smart drug delivery by several groups [17,18,19,20,21,22,23].

Aiming to improve drug efficacy, double or multiple response systems were designed to take advantage of the synergy between different stimulus actions. pH and reduction stimulus system, as two of the most efficient and significant methods aimed at tumor-targeting, can not only make corresponding morphological change to the pH value of the surrounding environment, but also present different states due to oxidation or a reduction [24,25]. Tian et al. prepared novel core cross-linked micelles as nano-templates for the wrap of an anti-cancer drug. The acrylic acid groups provided the pH-responsive characteristic and disulfide bonds broke down under both reductants of glutathione (GSH) and dithiothreitol (DTT), which had a faster and more efficient drug release [26]. Xiong et al. reported polymer micelles with pH-responsive segments and disulfide bonds in the backbone. In normal tissue conditions, the drug cumulative release percentage was only 23% after 72 h, but up to 90% after 72 h when introducing 10 mM GSH and acidic pH of an intracellular tumor cell condition [27].

**Scheme 1:**
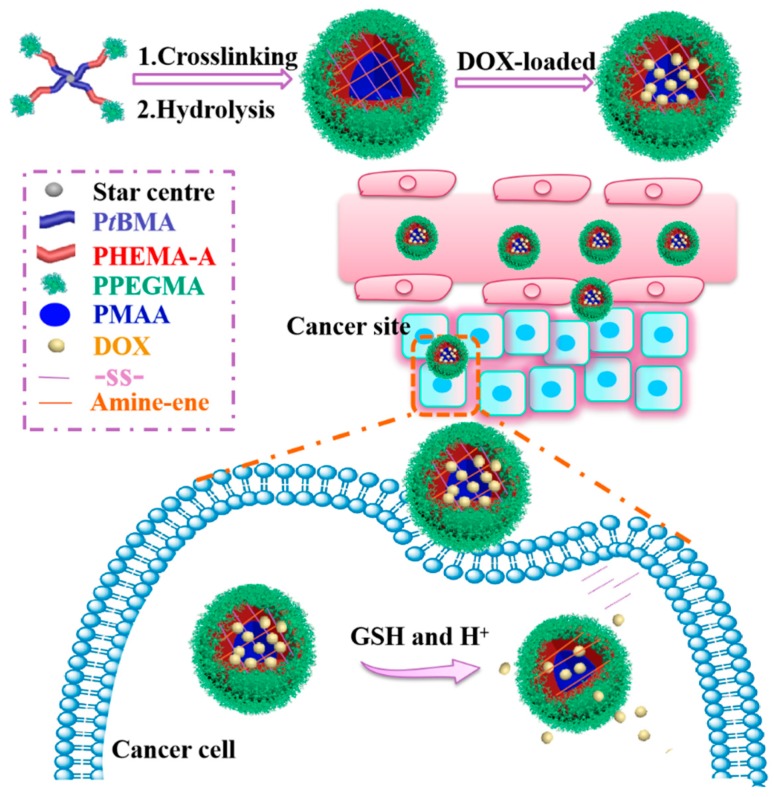
Schematic illustration of the preparation of pH/reduction sensitive DOX-loaded 4AS-PMAA-(PHEMA-SS~)-PPEGMA SCMs for cancer therapy.

In this present work, four-arm star-shaped copolymer poly(tertiary-butyl methacrylate)-*b*-poly(2-hydroxyethyl methacrylate)-*b*-poly(poly (ethylene glycol) methyl ether methacrylate) (4AS-P*t*BMA-PHEMA-PPEGMA) was designed and synthesized by activators regenerated by an electron transfer atom transfer radical polymerization (ARGET ATRP), which is followed by the Michael addition reaction and hydrolysis, and then self-assembled into three-layer disulfide-cross-linked SCMs. The Michael addition reaction herein is to react with the double bond on the triblock polymer with the amino group of cystamine and, thus, to form SCMs. The hydrophilic PPEGMA outer shell maintained the SCMs stability, the middle PHEMA layer was modified with double bond to introduce cross-linking sites, and the pH-sensitive poly (methacrylic acid) (PMAA) inner core was mainly used for anti-cancer drug doxorubicin (DOX) encapsulation and response to the tumor acidic condition. Because of the p*K*a of carboxylic acid groups in the PMAA core was around 5–6, it formed carboxylic acid (-COOH) at acidic pH and dissociate into carboxylate anions at higher pH. The electrostatic interaction between carboxyl acid groups on SCMs and amino groups in DOX molecules was beneficial to load more DOX at neutral conditions, while the electrostatic interaction was weakened, which triggers the fast release of DOX at tumor pH [28,29]. Meanwhile, disulfide-cross-linking could not only improve the SCMs stability to restrain the DOX released prematurely from micelles in normal tissues, but also break down under 10 mM of GSH in tumor cells to achieve fast DOX release. Thus, these SCMs could form a smart DOX release system with pH-triggered shrinking of PMAA core and reduction-driven degradation of the backbone (Scheme 1).

The micellar physicochemical properties, such as the DOX loading capacity, particle size, zeta potential, morphology, stability, and pH/reduction-sensitivity, were investigated by UV-vis spectrometry, dynamic light scattering (DLS), and transmission electron microscopy (TEM). The in vitro DOX release behavior was investigated by using fluorescence spectroscopy under pH and/or reduction stimulus-triggered conditions. Cytotoxicity was assessed by the 3-(4,5-dimethyl-2-thiazolyl)-2,5-diphenyl-2*H*-tetrazolium bromide (MTT) assay. Furthermore, by using fluorescence spectroscopy, kinetics and thermodynamics analysis were utilized to investigate details of the interaction of drug molecules released from polymer micelles, which hopes to find suitable nanocarriers for each anti-cancer drug.

## 2. Materials and Methods

### 2.1. Materials

Tertiary-butyl methacrylate (*t*BMA, TCI-EP), 2-hydroxyethyl methacrylate (HEMA, 99%, Aldrich), and poly (ethylene glycol) methyl ether methacrylate (PEGMA, *M*_n_ = 475 Da, 99%, Aldrich) were purified by passing through a neutral alumina column to remove the inhibitor. Pentaerythritol was dried under reduced pressure before use. Triethylamine (TEA), tetrahydrofuran (THF), dichloromethane (DCM), and toluene, all from J&K Scientific Ltd., were distilled from calcium hydride (CaH_2_) prior to use. Cystamine dihydrochloride (98%, Aldrich), 2-bromoisobutyryl bromide (98%, J&K Scientific Ltd.), 1,1,4,7,10,10-hexamethyltriethylenetetramine (HMTETA, 99%, J&K Scientific Ltd.), methacryloyl chloride (95%, J&K Scientific Ltd.), Pyrene (99%, J&K Scientific Ltd.), doxorubicin hydrochloride (DOX^.^HCl) (Wuhan Yuancheng Gongchuang Technology Co., Ltd.), trifluoroacetic acid (TFA), cupric bromide (CuBr_2_), stannous octoate (Sn(Oct)_2_), dimethylformamide (DMF), dimethyl sulfoxide (DMSO), *n*-hexane, and other reagents were used directly.

### 2.2. Characterizations

The copolymer structures were characterized using ^1^H NMR on a Bruker Avance III 400 MHz Nuclear Magnetic Resonance (NMR) spectrometer. The internal standard was tetramethylsilane (TMS). Solvents were CDCl_3_ or DMSO-*d*_6_. The SCMs sizes and zeta potential were determined by a Brookhaven BI-200SM dynamic light scattering (DLS) instrument equipped with a zetapals potential and particle size analyzer. Each sample was tested three times at a 632.8-nm He-Ne laser and the scattering angle of 90°. Transmission electron microscopy (TEM) was carried out on a Tecnai G220 TEM, and samples were fabricated by adding SCMs suspension (0.1 mg/mL) on copper grids.

### 2.3. Synthesis of Pentaerythritol Tetrakis (2-Bromoisobutyrate) [Br_4_]

Br_4_ was synthesized as the four hydroxy groups of pentaerythritol acylated by quadruple 2-bromoisobutyryl bromide. First, pentaerythritol (5.45 g, 40 mmol) and a magnetic stirring bar were charged into a flame-dried 250 mL Schlenk flask. Next, tetrahydrofuran (100 mL), triethylamine (22.24 mL, 160 mmol), and 2-bromoisobutyryl bromide (19.78 mL, 160 mmol) were injected dropwise under vigorous stirring into the flask orderly at 0 °C in an ice/water bath. This reaction process lasted for 5 h at 0 °C and another 24 h at 25 °C. Then, the received mixture solution was first filtered, evaporated, and diluted with DCM (80 mL), and then washed by HCl (3%, 200 mL), NaOH solution (2%, 200 mL), and deionized water (200 mL) in order. After that, the organic solution was obtained and dried through MgSO_4_. The crude product was obtained by rotary evaporation to remove solvent and precipitation with excess cold *n*-hexane followed by drying in a vacuum oven.

### 2.4. Synthesis of Four-Arm Star Copolymer 4AS-PtBMA-PHEMA-PPEGMA

Br_4_ was employed as the ARGET ATRP initiator to copolymerize *t*BMA, HEMA, and PEGMA [30,31]. First, the Br_4_ (442 mg, 0.6 mmol), CuBr_2_ (11.2 mg, 0.05 mmol), and a magnetic stirring bar were charged in a flame-dried 100 mL Schlenk flask and then evacuated the flask with argon three times. Then, anhydrous toluene (24 mL), *t*BMA (3.07 g, 21.6 mmol), and HMTETA (65 μL, 0.24 mmol) were injected into the flask with the following for 15 min stirring to form the Cu/HMTETA catalyst complex. Subsequently, Sn(Oct)_2_ (78 μL, 0.24 mmol) with toluene (1 mL) was added dropwise under argon protection to proceed with the reaction at 65 °C for 5 h under stirring. The reaction temperature was reduced to 55 °C and then the second monomer HEMA (1.87 g, 14.4 mmol), HMTETA (65 μL, 0.24 mmol), and Sn(Oct)_2_ (78 μL, 0.24 mmol) solution in toluene (1 mL) were injected to keep the reaction for another 12 h. Lastly, the third monomer PEGMA (6.84 g, 14.4 mmol), the HMTETA (65 μL, 0.24 mmol), and Sn(Oct)_2_ (78 μL, 0.24 mmol) solution in toluene (1 mL) was added orderly to continue the polymerization at 55 °C for 48 h. After the reaction, we diluted the reaction solution with THF (60 mL), which is followed by passing through a neutral alumina column and rotary evaporation. Lastly, the crude product was obtained by the precipitation with excess cold *n*-hexane followed by drying in a vacuum oven.

### 2.5. Synthesis of 4AS-PMAA-(PHEMA-SS~)-PPEGMA

The 4AS-PMAA-(PHEMA-SS~)-PPEGMA polymer was prepared in three steps. First, copolymer 4AS-P*t*BMA-PHEMA-PPEGMA (3.36 g, 0.2 mmol) was dissolved with 30 mL THF in a sealed flask and TEA (1.14 mL, 10 mmol) was then added by a syringe. The flask was then placed into an ice/water bath by adding methacryloyl chloride (shorten as A, 0.97 mL, 10 mmol) dropwise under continuous stirring. The acylation reaction was then proceeded at 0 °C for 2 h and at room temperature for another 22 h. Lastly, by passing a neutral alumina column, precipitated with excess of *n*-hexane, filtered off, the crude mixture is dried in vacuum to obtain acylation product 4AS-P*t*BMA-(PHEMA-A)-PPEGMA. Second, the dried acylation product (30 mg) was dissolved in 30 mL DMF and then poured into a dialysis bag (MWCO = 3.5 kDa). The bag was immersed in a phosphate buffer saline (PBS) buffer solution (50 mM, pH 9.0) by refreshing every 3 h for the first 18 h and every 8 h for another 16 h. Next, we poured out the reaction solution of the dialysis bag and adjusted the pH to 9.0 with NaOH solution (0.1 mol/L). The cross-linker of cystamine dihydrochloride (18.8 mg, 83.5 μmol) was then added to keep the reaction for another 24 h with vigorous stirring. Transferring the reaction solution into another dialysis bag and dialyzing against deionized water while refreshing every 3 h for 12 h to remove unreacted cross-linker. The 4AS-P*t*BMA-(PHEMA-SS~)-PPEGMA were collected by vacuum filtration and freeze-drying. Third, 4AS-P*t*BMA-(PHEMA-SS~)-PPEGMA (1 g) was dissolved in 20 mL DCM and reacted with TFA (1.5 mL) at 0 °C for 30 min and then at 25 °C for another 4 h to hydrolyze the ester groups [32,33,34]. The final target copolymer 4AS-PMAA-(PHEMA-SS~)-PPEGMA was obtained after removing all solvent and drying under vacuum overnight.

### 2.6. Preparation of Blank and DOX-Loaded SCMs

The blank and DOX-loaded 4AS-PMAA-(PHEMA-SS~)-PPEGMA SCMs were obtained by self-assembly using the dialysis method. First, DOX·HCl (14 mg or 28 mg) as a model drug was dissolved in DMSO (20 mL), which was followed by adding TEA (2-fold molar of the DOX·HCl) dropwise and stirred for 4 h to form DOX base solution. Next, polymer 4AS-PMAA-(PHEMA-SS~)-PPEGMA (60 mg) was dissolved into another DMSO (20 mL, and 40 mL for blank SCMs), and then the prepared DOX base solution was added. Subsequently, the solutions were poured into dialysis bags (MWCO = 3.5 kDa) and dialyzed against deionized water refreshed every 4 h for 24 h to remove DMSO. After dialysis, the micelle solutions were filtered with the membrane (0.45 μm pore) to remove free DOX, and obtained dried pure products after freeze-drying. The particle sizes and morphologies of the blank SCMs and DOX-loaded SCMs were determined by DLS and TEM, and the drug loading capacities of DOX-loaded SCMs were analyzed by measuring a TU-1901 UV-vis spectrophotometer (Beijing Purkinje General Instrument Co., Ltd., Beijing, China) at 480 nm to obtain drug loading content (DLC) and drug loading efficiency (DLE), which were calculated using the following equation.

(1) $$\mathrm{DLC}\left(\mathrm{wt}\%\right)=\frac{\mathrm{weight}\;\mathrm{of}\;\mathrm{loaded}\;\mathrm{drug}}{\mathrm{total}\;\mathrm{weight}\;\mathrm{of}\;\mathrm{polymer}\;\mathrm{and}\;\mathrm{loaded}\;\mathrm{drug}}\times 100$$

(2) $$\mathrm{DLE}\left(\%\right)=\frac{\mathrm{weight}\;\mathrm{of}\;\mathrm{loaded}\;\mathrm{drug}}{\mathrm{weight}\;\mathrm{of}\;\mathrm{drug}\;\mathrm{in}\;\mathrm{feed}}\times 100$$

### 2.7. Stability and pH/Reduction-Sensitivity Properties of SCMs

The stability of SCMs was determined by diluting with extensive deionized water and adding organic solvent of DMF. One sample of SCMs aqueous solution (1 mg/mL) was diluted with 1000-fold volume of deionized water, while the other was added four-fold volume of DMF. After stirring for 24 h, the changes of related micelle sizes were measured by DLS.

The pH-driven and GSH-driven transformation in micelle sizes and zeta potentials were investigated by DLS at a temperature of 25 °C. Four samples of SCMs solution (1 mg/mL) were adjusted to different pH values with PBS or acetate buffer solutions under GSH or no GSH conditions at (1) pH 7.4, (2) pH 5.0, (3) pH 7.4 with 10 mM GSH, and (4) pH 5.0 with 10 mM GSH.

### 2.8. In Vitro DOX Release and Thermodynamic Studies

Since the drug DOX had fluorescence properties with the optimal excitation and emission wavelength of E_x_ = 465 nm and E_m_ = 558 nm, respectively, the in vitro DOX release behavior under different simulated conditions was examined by using a Hitachi F-7000 fluorescence spectrophotometer from 480 to 720 nm of emission after excitation at 465 nm with the slit width at 5 nm for both emission and excitation.

DOX-loaded SCMs (5 mg) were re-dispersed in a PBS or acetate buffer solutions (5 mL) under GSH or no GSH conditions: (1) pH 7.4, (2) pH 5.0, (3) pH 7.4 with 10 mM GSH, and (4) pH 5.0 with 10 mM GSH. These DOX-loaded SCMs solutions were charged into dialysis bags (MWCO = 3.5 kDa) and then immersed into 95 mL of the same buffer solution in an incubation shaker (Shanghai Yiheng THZ-100) at the temperature of 310 K with 100 rpm rotation speed. At the pre-determined time intervals, 4-mL samples were collected for the DOX release dynamics study by fluorescence spectroscopy measurement, while another 4 mL of fresh buffer solution was added to keep the dispersion volume constant. All samples were analyzed in triplicates to produce the final release curves.

In the thermodynamic study, triplicate of the above DOX-loaded SCM solutions at different simulated conditions ((1) pH 7.4, (2) pH 5.0, (3) pH 5.0 + 10 mM) were placed in a shaker with 100 rpm of rotation speed at the temperatures of 304 K, 310 K, and 314 K, respectively. At the pre-determined time intervals, 4 mL samples were taken for measurement while another with the same volume of fresh buffer was added into the system.

### 2.9. Cytotoxicity Assay

The in vitro cytotoxicity was analyzed by the MTT assay. The cultured HepG2 cells (1 × 10^4^ cells/well) were inoculated into 96-well cell plates with Dulbecco’s Modified Eagle’s medium (DMEM) medium and cultured at 5% CO_2_ and 37 °C for 24 h. Then, the free DOX, blank SCMs, and DOX-loaded SCMs were added to replace the old medium and cultured for 48 h (or 24 h). Next, fresh DMEM (180 mL) was added to replace the culture medium, which is followed by adding MTT solution (20 μL, 5 mg/mL in PBS buffer), and incubated for another 4 h. After removing the unreacted MTT solution, we added DMSO (200 μL) to dissolve MTT crystals. The 96-well plates were placed in a shaking bed at 37 °C and oscillated for 15 min, and the absorbance was determined via a microplate reader at 570 nm.

## 3. Results and Discussion

### 3.1. Synthesis and Characterization of 4AS-PMAA-(PHEMA-SS~)-PPEGMA

**Scheme 2:**
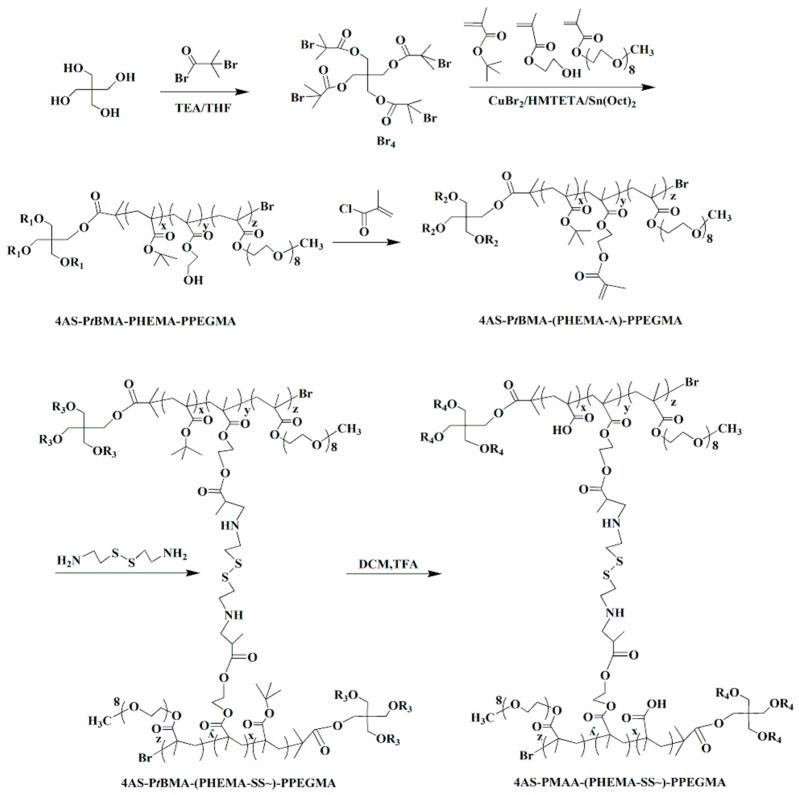
Synthetic route of the 4AS-PMAA-(PHEMA-SS~)-PPEGMA.

The copolymer 4AS-PMAA-(PHEMA-SS~)-PPEGMA was synthesized using the combination of ARGET ATRP, Michael addition reaction, and hydrolysis method (Scheme 2). In the first step, the functional initiator Br_4_ was obtained by using pentaerythritol reacted with 2-bromoisobutyryl bromide at 25 °C. Then, the ARGRT ATRP of *t*BMA, HEMA, and PEGMA proceeded successively with Br_4_ as the initiator, CuBr_2_/HMTETA as the catalyst system, and Sn(Oct)_2_ as a reducer to obtain 4AS-P*t*BMA-PHEMA-PPEGMA. Next, the cross-linker of cystamine dihydrochloride was introduced to build a cross-linked structure with the copolymer using the Michael addition reaction. Lastly, the copolymer 4AS-PMAA-(PHEMA-SS~)-PPEGMA was synthesized by hydrolysis to remove the tertiary butyl groups from *t*BMA units.

**Figure 1:**
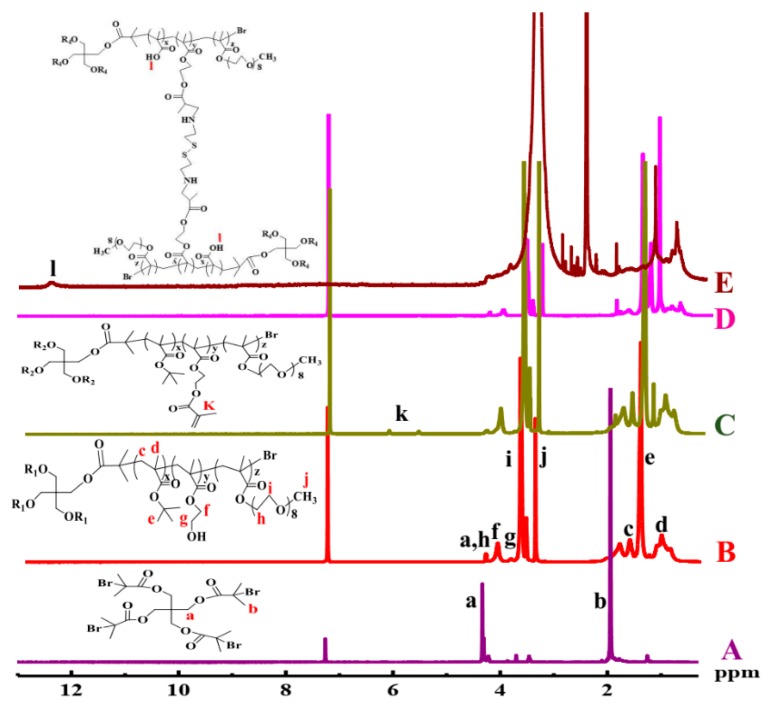
^1^H NMR spectra of (**A**) Br_4_, (**B**) 4AS-PtBMA-PHEMA-PPEGMA, (**C**) 4AS-PtBMA-(PHEMA-A)-PPEGMA, and (**D**) 4AS-PtBMA-(PHEMA-SS~)-PPEGMA in CDCl_3_, and (**E**) 4AS-PMAA-(PHEMA-SS~)-PPEGMA in DMSO-*d*_6_.

The chemical structure of copolymer 4AS-PMAA-(PHEMA-SS~)-PPEGMA and the precursors were measured by ^1^H NMR (Figure 1). As shown in Figure 1A, the signals at 1.93 (b) and 4.33 ppm (a) were ascribed to -C(CH_3_)_2_-Br and -COO-CH_2_- of pentaerythritol tetrakis (2-bromoisobutyrate). Figure 1B was the ^1^H NMR spectrum of copolymer 4AS-P*t*BMA-PHEMA-PPEGMA. The signals at 0.8–1.2 (d), 1.41 (e), and 1.62 ppm (c) were due to -CCH_3_, -(CH_3_)_3_-, and -CH_2_- protons of the P*t*BMA block, respectively, while the signals at 3.84 (g) and 4.09 ppm (f) were the coterminous two methylene protons of -CH_2_CH_2_- in the HEMA unit. The characteristic peaks of PPEGMA at 3.38 (j), 3.66 (i), and 4.30 ppm (h) were ascribed, respectively, to -OCH_3_, -OCH_2_-CH_2_O-, and -COO-CH_2_-protons, respectively. From the calculation of the integration ratio values of signal (e) to (b) (*I*_e_/*I*_b_), signal (f) to (e) (*I*_f_/*I*_e_) and signal (j) to (f) (*I*_j_/*I*_f_), respectively, the degree of polymerization (DP) of P*t*BMA (x = 8.1), PHEMA (y = 4.4), PPEGMA (z = 5.0) were obtained, and also the *M*_n_ values = 16,801 of 4AS-P*t*BMA-PHEMA-PPEGMA. The *M*_n_ and DP were consistent with the theoretical values (*M*_n, theory_ = 20,044), which indicated 4AS-P*t*BMA-PHEMA-PPEGMA has been synthesized successfully.

The representative ^1^H NMR spectra of 4AS-PtBMA-(PHEMA-A)-PPEGMA, 4AS-PtBMA-(PHEMA-SS~)-PPEGMA, and 4AS-PMAA-(PHEMA-SS~)-PPEGMA were shown in Figure 1C–E, respectively. Clearly, there appeared two proton signals at 5.61 and 6.16 ppm (k) in the Figure 1C, due to the C=C group introduced. The signals of C=C group disappeared due to the formation of crosslinking structure (Figure 1D), and the M_n_ values of 4AS-PtBMA-(PHEMA-SS~)-PPEGMA was 37,685 Da. With being hydrolyzed, the tert-butyl group (1.41 ppm) (e) disappeared and the carboxyl group (12.30 ppm) (l) emerged, which could be found in Figure 1E. Thus, all the data demonstrated that the final product 4AS-PMAA-(PHEMA-SS~)-PPEGMA has been synthesized successfully with the M_n_ values of 34,101 Da.

### 3.2. Properties of Blank and DOX-Loaded SCMs

**Figure 2:**
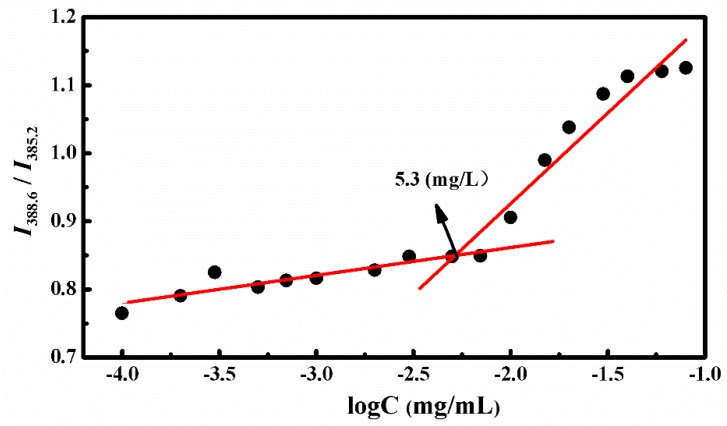
Plot of intensity ratios (I_388.6_/I_385.2_) as a function of 4AS-PMAA-(PHEMA-SS~)-PPEGMA concentrations.

The amphiphilic star copolymer can be self-assembled into three layers of SCMs in aqueous solution when the concentration reached the critical micelle concentration (CMC) value. The CMC was obtained by the fluorescence technique with pyrene as the probe. Since the pyrene probe could migrated from the aqueous solution to the hydrophobic core of the micelles while increasing the copolymer concentration, we measured the emission spectra of polymer/pyrene solution and received the changes of the peak from 385.2 to 388.6 nm at an excitation wavelength of 340 nm. Figure 2 showed the ratios of I_388.6_ to I_385.2_ against different polymer concentrations, and the interception of two straight lines was CMC, which was valued at 5.3 mg/L. This suggested that the amphiphilic polymers could self-assemble into stable micelles even at low concentrations.

**Figure 3:**
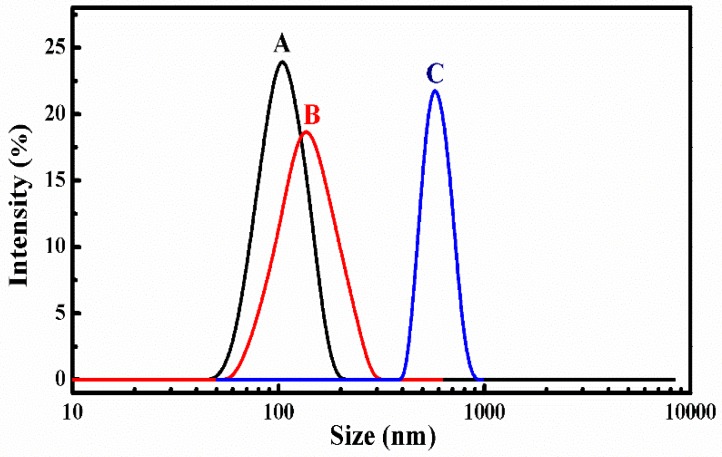
Size distribution of blank SCMs: (**A**) before, (**B**) after 1000-fold water, and (**C**) four-fold DMF dilution.

The stability of SCMs was investigated by diluting with extensive water and adding organic solvent of DMF to measure the size and distribution changes by DLS, as shown in Figure 3 [12,35]. First, the SCMs still existed even after diluting with a 1000-fold volume of deionized water (C < CMC) and the particle size was slightly increased with a low polydispersity index (PDI). Although the organic solvent of DMF could improve the solubility to all chain segments of SCMs, the SCMs only swelled to a larger particle size, but was not disassembled into small unimers. This indicated good stability to avoid premature DOX burst release before arriving at the target sites.

**Figure 4:**
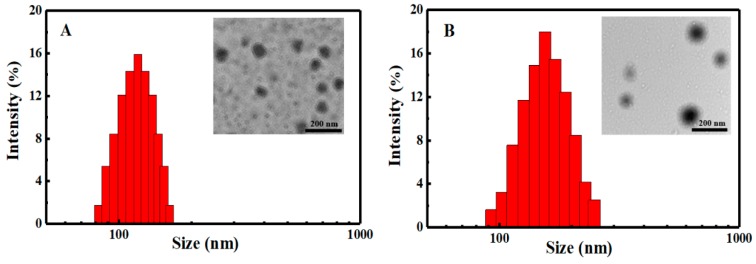
DLS and TEM of (**A**) blank and (**B**) DOX-loaded SCMs.

Particle sizes and zeta potentials of the blank SCMs and DOX-loaded SCMs with different DLC and DLE dispersed in deionized water (pH = 6.9) were investigated by DLS and UV-vis, as shown in Figure 4 and Table 1. At a neutral solution, the carboxylic acid groups on the PMAA segments could be ionized into carboxylate anions, while DOX molecules protonated as cations (pK_a_ of 8.25) [36]. Therefore, through an electrostatic interaction, DOX was wrapped onto the SCMs. With the increase of fed DOX, the DLC, which were calculated to be 14.73% and 24.97%, increased accordingly, while the DLE decreased from 74.05% to 71.32% as the relative saturated capacity of solubilizing DOX in the micellar cores. All particle sizes of the blank SCMs and DOX-loaded SCMs were below 180 nm with narrow unimodal distribution (PDI < 0.2), which indicated a good physical property of the SCMs. Moreover, particle sizes of DOX-loaded SCMs were relatively larger than that of blank SCMs, while size distributions and zeta potentials had only small changes, which suggests that DOX was incorporated into the SCMs effectively and then increased the particle sizes to a certain extent.

TEM analysis (Figure 4) revealed that the blank SCMs and DOX-loaded SCMs both possessed a spherical morphology, and the size of blank SCMs was about 100 nm while the DOX-loaded SCMs was about 140 nm. These results were in agreement with those determined by DLS.

### 3.3. pH- and GSH-Driven In Vitro DOX Release Behavior

Since the DOX released from the SCMs to aqueous solution could be monitored by the change of fluorescence spectroscopy intensity, we investigated pH-driven and GSH-driven in vitro release of DOX using fluorescence spectroscopy.

**Figure 5:**
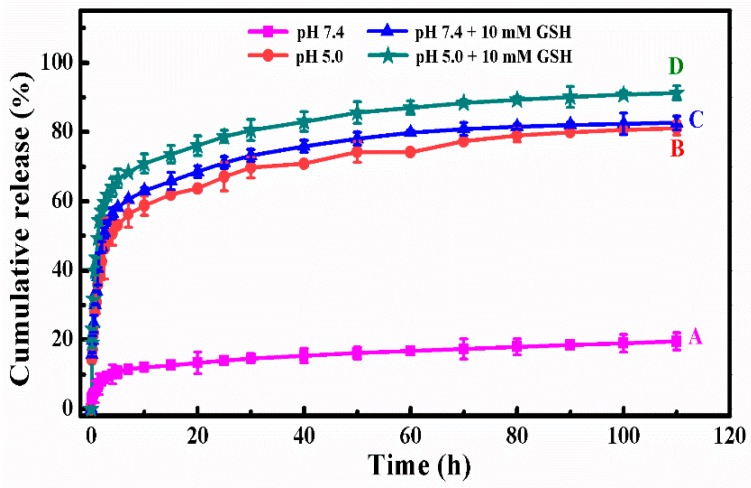
The in vitro DOX release profiles of DOX-loaded SCMs at different simulated conditions: (**A**) pH 7.4, (**B**) pH 5.0, (**C**) pH 7.4 + 10 mM GSH, and (**D**) pH 5.0 + 10 mM GSH.

Figure 5 showed the in vitro DOX release behavior of DOX-loaded SCMs under different simulated conditions at the temperature of 310 K. Under the normal physiological condition (pH 7.4, Figure 5A), the DOX cumulative release percentages were only 11.4% in the initial 7 h and 19.5% within 110 h. After a certain time, the cumulative release reached a plateau, which suggested that the release of DOX came into the last stage.

In a more acidic condition (pH 5.0) shown in Figure 5B, there was a fast DOX release in the initial time and it then reached a plateau as time went on. The DOX cumulative release percentage improved to 48.6% in the initial 3 h and then reached 81.1% within 110 h. Under this acidic condition, the higher cumulative release was due to the electrostatic interaction between the DOX and micelles decreasing [37]. Moreover, the dissolubility of DOX in mildly acidic solution was relative higher than that in neutral media and the deionization of carboxylic acid groups in MAA units resulted in the shrinkage of the micelles’ structure to squeeze the entrapped DOX released from polymer micelles [38]. The results indicated the pH-sensitive controlled release performance of the SCMs.

The reductive value in tumor sites is about 100–1000 times higher than that in the normal tissue, which is another apparent physiological difference besides pH value. Therefore, our reductive-sensitive SCMs could also realize the “on-demand” anti-cancer drug release in the tumor microenvironment. Figure 5C,D showed the DOX release behavior at simulated conditions of pH 7.4 with 10 mM GSH and pH 5.0 with 10 mM GSH. After introducing 10 mM of GSH, the disulfide bonds in the backbone of the cross-linked polymer micelles were broken down and the micellar architecture was disassembled, which led to much faster DOX release than those without GSH. The DOX cumulative release percentages were 53.9% and 61.9% in the initial 3 h, and then reached 82.6% and 91.3% within 110 h, respectively. In addition, the DOX release percentage at a pH of 5.0 was higher than that at a pH of 7.4.

### 3.4. DOX Release Kinetics

**Figure 6:**
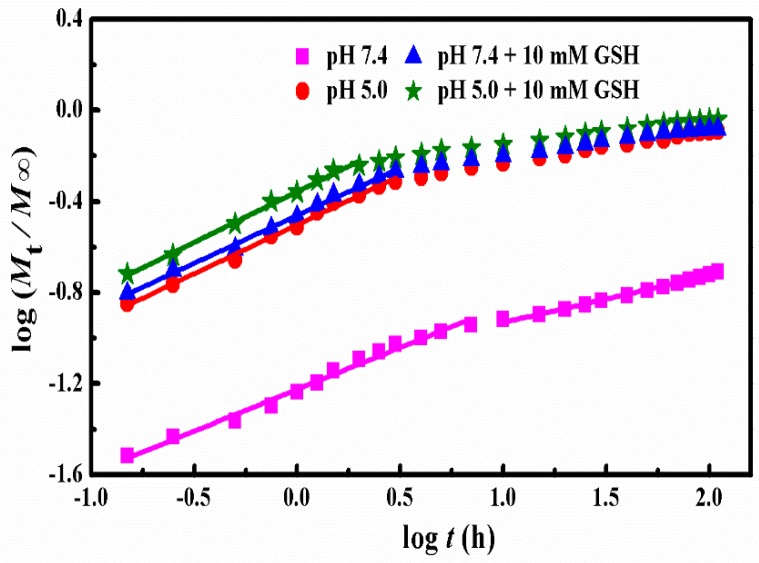
Plots of log (*M_t_*/*M_∞_*) versus log t for DOX release from SCMs under different conditions.

The drug release mechanism is complex and analyzed currently by the following semi-empirical equations [39,40,41].

(3) $$\frac{M_{t}}{M_{\infty }}=k\;t^{n}$$

(4) $$log\left(\frac{M_{t}}{M_{\infty }}\right)=nlog\;t+nlog\;k$$

where *n* represents the release exponent suggesting the drug release mechanism and *k* represents a constant incorporating the structural and geometric characteristics of the copolymer matrix. *M_t_* and *M_∞_* represent the absolute cumulative amounts of drug release at time *t* and infinite time, respectively. As for the spherical nanoparticles, the n value of 0.43 is Fickian diffusion while 0.85 is non-Fickian diffusion. In addition, *n* < 0.43 corresponds to the diffusion and erosion control while 0.43 < *n* < 0.85 is attributed to the anomalous transport mechanism [42]. The k and n were gained from the plots of log (*M_t_*_/_*M_∞_*) versus logt at different simulated conditions (Figure 6) and the obtained results were listed in Table 2.

Clearly, the release of DOX from SCMs under all stimulated conditions presented two stages with good linearity. Under the conditions of pH 7.4, the n values of two stages were lower than 0.43, which suggested that the whole release process was for diffusion and erosion control. In a more acidic condition (pH 5.0), the n value of the first stage was near 0.43, which indicated the DOX release was Fickian diffusion. Higher dissolubility of DOX in mildly acidic solution and the shrink of the micelles structure because of the deionization of carboxylic acid groups in MAA units led to faster DOX released from polymer micelles. Moreover, after introducing 10 mM of GSH, the stage II that began from 7 h declined to 3 h at pH 7.4 and from 3 to 2 h at low acid pH 5.0. This could be attributed to the destruction of the cross-linking under 10 mM GSH condition, which produced a more rapid release of the DOX molecules. The *n* values of the first stage was around 0.43 and was higher than 0.43, which was ascribed to Fickian diffusion and anomalous transport mechanism, respectively. The *k* values increased visibly as the pH decreased with or without 10 mM GSH, which suggested the DOX release rate increased sharply with the decrease of pH values and the addition of GSH. In the second stage, the cumulative drug release percentage (*M_t_*/*M_∞_*) was much higher than 60% for acid and reduction conditions. Therefore, the model was no longer applicable for the mechanism analysis [27,43,44]. In addition, there is no n and *k* values for these three samples in Figure 6 and Table 2.

The results of the pH-triggered and GSH-triggered in vitro DOX release behavior of DOX-loaded SCMs prove that the SCMs could form a good drug delivery system. On the one hand, the SCMs could efficiently restrain the premature release of DOX in bloodstream and normal tissues. On the other hand, when arriving at the target sites, the SCMs could release DOX rapidly by introducing a pH/reduction dual stimulus response, which was more efficient to enhance the faster release of DOX compared with those of single pH-responsive or reduction-responsive systems.

### 3.5. DOX Release Thermodynamics

**Figure 7:**
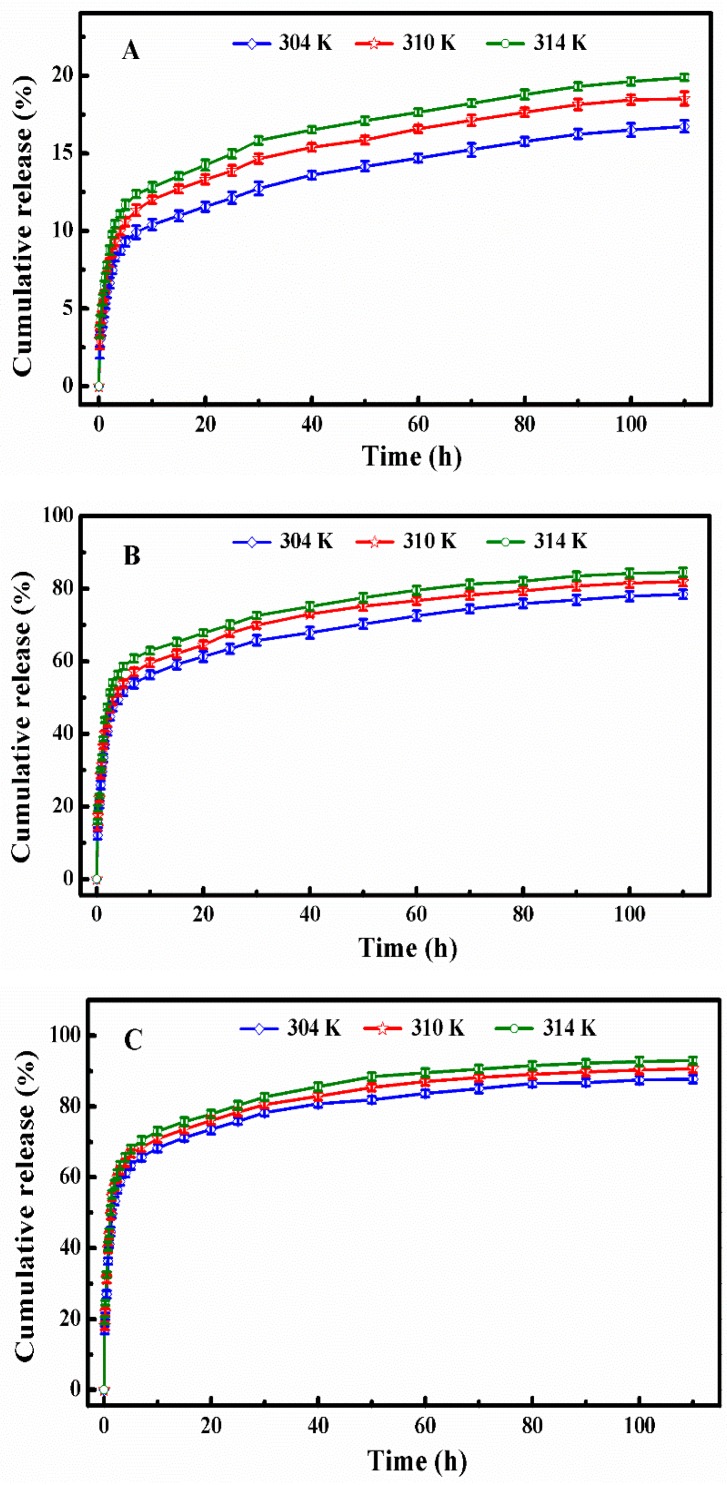
The in vitro DOX release profiles of DOX-loaded SCMs at the temperatures of 304, 310, and 314 K under different simulated conditions: (**A**) pH 7.4, (**B**) pH 5.0, and (**C**) pH 5.0 + 10 mM.

In general, the driven forces between the anti-cancer drug and micelles include Van der Waals force, hydrogen bonding, electrostatic, and hydrophobic interactions [45]. Herein, we explored the thermodynamic study to analyze the interactions between DOX and polymer micelles in the DOX release process using fluorescence spectroscopy, and the samples were prepared at the temperatures of 304 K, 310 K, and 314 K under different simulated conditions (Figure 7). The thermodynamic parameters of enthalpy change (Δ*H*), entropy change (Δ*S*), and free energy change (Δ*G*), calculated from Equation (5) to Equation (7), were investigated to further understand the DOX release process.

(5) $$K_{d}=\frac{C_{ap}}{C_{mp}}=\frac{C_{ap}}{m_{DOX}(100-E_{ap})/V_{0}}$$

(6) $$\ln\;K_{d}=-\frac{\Delta H}{RT}+\frac{\Delta S}{R}$$

(7) ΔG=ΔH−TΔS

**Figure 8:**
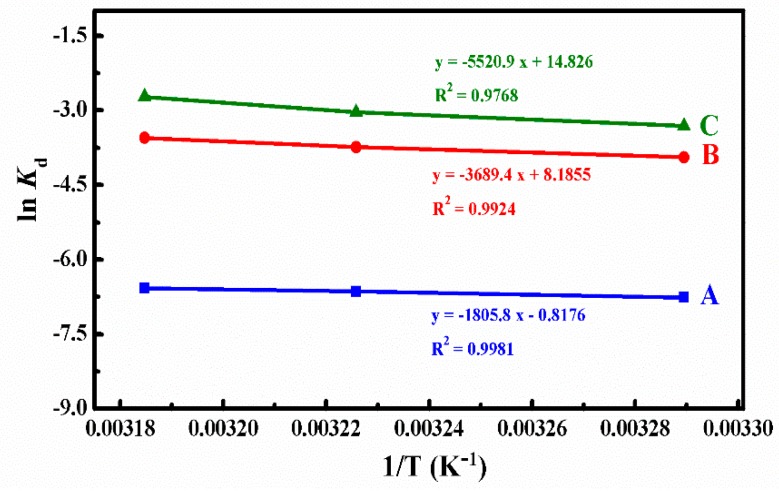
The plots of ln *K*_d_ versus 1/T at different simulated conditions: (**A**) pH 7.4, (**B**) pH 5.0, and (**C**) pH 5.0 + 10 mM.

*K*_d_ represents the release constant at the temperature of T, and we can calculate it using Equation (5) on the base of the data in Figure 7. Where *C*_ap_ and *C*_mp_ are the concentrations of DOX (g/L) in the aqueous solution and micelles of the *i*th sample after the DOX cumulative release reached the plateau, *E*_ap_ are the DOX cumulative release percent until the *i*th sample, *R* is the universal gas constant (8.3145 J·mol^−1^·K^−1^), and the Δ*H* and Δ*S* could be determined from the slope and intercept of the plot of ln *K*_d_ versus 1/T (Figure 8). All thermodynamics parameters were summarized in Table 3.

From the calculated results summarized in Table 3, we could find that the Δ*G* values were positive at all different simulated conditions, which meant that the DOX release process was not spontaneous. The Δ*G* absolute values were around 7.20–17.15 kJ/mol and belonged to the range of free energy of physical release, which indicated that the DOX release process was a physically driven release process. In the systems of pH 5.0 and pH 5.0 + 10 mM GSH, Δ*G* decreased with the increase of temperature, which indicated that higher temperature was in favor of the spontaneous of DOX release from micelles. Therefore, the absorbed heat accelerated the DOX release.

The Δ*H* values represented the main forces in the process of DOX release. At different simulated conditions, the Δ*H* values were around 15.01–45.90 kJ/mol, which belonged to the energy scope of electrostatic interaction. This suggested that the DOX release process was controlled mainly by the force of an electrostatic interaction between the functional groups of DOX and polymer micelles. Because of the broken disulfide bond, the ΔH value was much larger at the simulated condition of pH 5.0 with 10 mM GSH, which represents the existence of a chemical bond force. Meanwhile, the Δ*H* values were positive at different simulated conditions, which meant that the DOX release process was endothermic and needed a certain quantity of heat to destroy the electrostatic interaction for DOX release.

The value of Δ*S* revealed the degree of chaos in the solution system. At pH 7.4, Δ*S* was negative. The degree of freedom of the system decreased because the DOX was confined to the micelles. The changes of the Δ*S* value was small at pH 7.4, which indicates that the DOX release was mainly an enthalpically-driven process. At the conditions of pH 5.0 and pH 5.0 with 10 mM GSH, the values of Δ*S* were positive since hydrophobic interaction led to the shrinkage of polymer micelles to squeeze much of the entrapped DOX released from polymer micelles into the water solution, and, thus, increased the chaotic degree of the solution system. As the Δ*S* changed values were rather large, the DOX release processes were mainly entropically driven at the two simulated conditions.

### 3.6. Cytotoxicity Analysis

**Figure 9:**
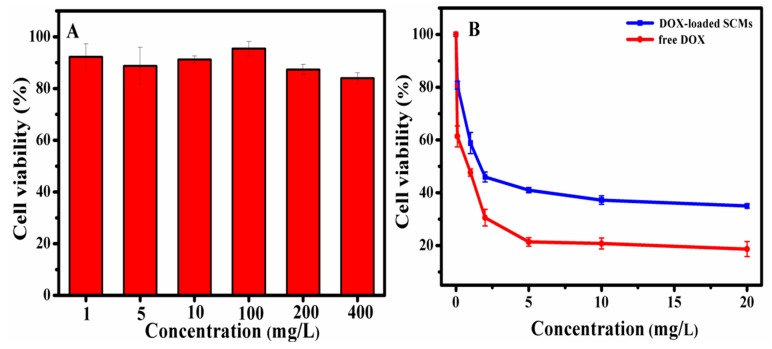
The cytotoxicity of (**A**) blank SCMs, (**B**) DOX-loaded SCMs, and free DOX against HepG2 cells after 48 h.

The MTT assay was used to determine the cytotoxicity of the blank SCMs and DOX-loaded SCMs against HepG2 cells. As the cytotoxicity of blank SCMs shown in Figure 9A, the blank SCMs exhibited great nontoxic property, since the viability of cells was higher than 84.0%, even when the concentration of the blank SCMs rose to 400 mg/L. After introducing the anti-cancer drug DOX (Figure 9B), the viability of cells incubated with the DOX-loaded SCMs was 100% to 35.0% at the concentration range of 0 to 20 mg/L, while the viability of cells incubated with free DOX decreased to 16.0% at 20 mg/L concentration. Since the SCMs were sensitive to acidic pH and GSH, the cumulative DOX release from the DOX-loaded SCMs reached 83% in 48 h (Figure 5) but did not complete the release, and, therefore, the DOX-loaded SCMs were shown to be relatively less toxic than free DOX [36].

## 4. Conclusions

A novel copolymer 4AS-PMAA-(PHEMA-SS~)-PPEGMA with disulfide bonds on the backbone was designed and synthesized. The amphiphilic copolymer had a low CMC value (5.3 mg/L) and could self-assemble into three layers SCMs with PMAA inner core used for DOX encapsulation and responding to an acidic pH value. The SCMs possessed superior stability against extensive water dilution and organic solvent DMF. Fluorescence spectroscopy was employed to investigate the DOX release behaviors under different physiological conditions. The data revealed that the DOX-loaded SCMs had lower DOX cumulative release percentages in normal physiological conditions, but higher DOX cumulative release percentages with rapid release when placed in tumor sites with mildly acidic pH and 10 mM of GSH. This suggested that the SCMs could not only improve the anti-cancer efficiency, but also reduce their toxic side effect to the normal tissues. After the thermodynamic analysis, we found that the DOX release was endothermic and not a spontaneous process. At pH 7.4, the DOX release process was enthalpically favored and controlled mainly by the force of an electrostatic interaction. At pH 5.0, the DOX release process was entropy-driven and the interactions between the SCMs and DOX molecules underwent an electrostatic interaction and hydrophobic interactions. At a pH of 5.0 with a 10 mM GSH condition, the DOX release process was entropy-driven and controlled mainly by the forces of an electrostatic interaction, a chemical bond, and a hydrophobic interaction. The blank SCMs exhibited high cell viability, while the DOX-loaded SCMs owned well cytotoxicity. All the results revealed that these SCMs could form a good drug delivery system to control anti-cancer drug release, and the drug release kinetic and thermodynamic analysis demonstrated the feasibility to serve as an indication for the interaction of drug molecules released from polymer matrices, which provide more details to help us understand the drug release process more accurately.

## Figures and Tables

**Table 1:** D_h_, PDI, zeta potential, DLC, and DLE of blank and DOX-loaded SCMs.

Sample	DOX/Polymer (mg/mg)	DLC (%)	DLE (%)	*D*_h_ (nm)	PDI	Zeta Potential (mV)
Micelle-1	0/60			122.9	0.147	−14.41
Micelle-2	14/60	14.73	74.05	160.4	0.173	−20.22
Micelle-3	28/60	24.97	71.32	172.4	0.150	−22.35

**Table 2:** Fitting parameters of the in vitro DOX release mechanism at different conditions.

Condition	*n*_1_	*k*_1_	*R* ^2^	*n*_2_	*k*_2_	*R* ^2^
pH 7.4 ^a^	0.3668	0.0596	0.9890	0.2060	0.0728	0.9927
pH 5.0 ^b^	0.4292	0.3143	0.9941	-	-	-
pH 7.4 + GSH ^c^	0.4190	0.3453	0.9965	-	-	-
pH 5.0 + GSH ^d^	0.4453	0.4391	0.9952	-	-	-

^a^ Stage I is 0–7 h, stage II is 7–110 h. ^b,c^ Stage I is 0–3 h, stage II is 3–110 h. ^d^ Stage I is 0–2 h, stage II is 2–110 h.

**Table 3:** Thermodynamic parameters for the interaction of DOX and 4AS-PMAA-(PHEMA-SS~)-PPEGMA SCMs in the DOX release process at 304 K, 310 K, and 314 K at 100 h.

Condition	T (K)	∆*H* (kJ/mol^−1^)	R^2^	∆*S* (J/mol^−1^/k^−1^)	∆*G* (kJ/mol^−1^)
pH 7.4	304	15.01	0.9981	−6.80	17.08
	310				17.12
	314				17.15
pH 5.0	304	30.68	0.9924	68.06	9.99
	310				9.58
	314				9.31
pH 5.0 + 10 mM GSH	304	45.90	0.9768	123.27	8.43
	310				7.69
	314				7.20

## References

[1] Jiang J., Li J., Zhou B., Niu C., Wang W., Wu W., Liang J. (2019). Fabrication of Polymer Micelles with Zwitterionic Shell and Biodegradable Core for Reductively Responsive Release of Doxorubicin. Polymers.

[2] Xiong D., Zhang X., Peng S., Gu H., Zhang L. (2018). Smart pH-sensitive micelles based on redox degradable polymers as DOX/GNPs carriers for controlled drug release and CT imaging. Colloids Surf. B Biointerfaces.

[3] Choi Y.I., Choi E.-S., Mun K.H., Lee S.G., Lee S.J., Jeong S.W., Lee S.W., Kim H.-C. (2019). Dual-responsive Gemini Micelles for Efficient Delivery of Anticancer Therapeutics. Polymers.

[4] Qiu M., Ouyang J., Sun H., Meng F., Cheng R., Zhang J., Cheng L., Lan Q., Deng C., Zhong Z. (2017). Biodegradable Micelles Based on Poly(ethylene glycol)-b-polylipopeptide Copolymer: A Robust and Versatile Nanoplatform for Anticancer Drug Delivery. ACS Appl. Mater. Interfaces.

[5] Zhao Y., Wu Y., Xue B., Jin X., Zhu X. (2019). Novel target NIR-fluorescent polymer for living tumor cell imaging. Polym. Chem..

[6] Oerlemans C., Bult W., Bos M., Storm G., Nijsen J.F.W., Hennink W.E. (2010). Polymeric micelles in anticancer therapy: Targeting, imaging and triggered release. Pharm. Res..

[7] Zhong G., Yang C., Liu S., Zheng Y., Teo J.Y., Lou W., Bao C., Cheng W., Tan J.P., Gao S. (2019). Polymers with distinctive anticancer mechanism that kills MDR cancer cells and inhibits tumor metastasis. Biomaterials.

[8] Lin W., Yao N., Li H., Hanson S., Han W., Wang C., Zhang L. (2016). Co-Delivery of Imiquimod and Plasmid DNA via an Amphiphilic pH-Responsive Star Polymer that Forms Unimolecular Micelles in Water. Polymers.

[9] Ge Z., Liu S. (2013). Functional block copolymer assemblies responsive to tumor and intracellular microenvironments for site-specific drug delivery and enhanced imaging performance. Chem. Soc. Rev..

[10] Li Y., Xiao K., Zhu W., Deng W., Lam K.S. (2014). Stimuli-responsive cross-linked micelles for on-demand drug delivery against cancers. Adv. Drug Deliv. Rev..

[11] Maiti C., Parida S., Kayal S., Maiti S., Mandal M., Dhara D. (2018). Redox-Responsive Core-Cross-Linked Block Copolymer Micelles for Overcoming Multidrug Resistance in Cancer Cells. ACS Appl. Mater. Interfaces.

[12] Xiong D., Yao N., Gu H., Wang J., Zhang L. (2017). Stimuli-responsive shell cross-linked micelles from amphiphilic four-arm star copolymers as potential nanocarriers for “pH/redox-triggered” anticancer drug release. Polymer.

[13] Li L., Li D., Zhang M., He J., Liu J., Ni P. (2018). One-Pot Synthesis of pH/Redox Responsive Polymeric Prodrug and Fabrication of Shell Cross-Linked Prodrug Micelles for Antitumor Drug Transportation. Bioconjug. Chem..

[14] Xue S., Gu X., Zhang J., Sun H., Deng C., Zhong Z. (2018). Construction of Small-Sized, Robust, and Reduction-Responsive Polypeptide Micelles for High Loading and Targeted Delivery of Chemotherapeutics. Biomacromolecules.

[15] Talelli M., Barz M., Rijcken C.J., Kiessling F., Hennink W.E., Lammers T. (2015). Core-crosslinked polymeric micelles: Principles, preparation, biomedical applications and clinical translation. Nano Today.

[16] Su L., Li R., Khan S., Clanton R., Zhang F., Lin Y.-N., Song Y., Wang H., Fan J., Hernandez S. (2018). Chemical Design of Both a Glutathione-Sensitive Dimeric Drug Guest and a Glucose-Derived Nanocarrier Host to Achieve Enhanced Osteosarcoma Lung Metastatic Anticancer Selectivity. J. Am. Chem. Soc..

[17] Sun H., Zhang Y., Zhong Z. (2018). Reduction-sensitive polymeric nanomedicines: An emerging multifunctional platform for targeted cancer therapy. Adv. Drug Deliv. Rev..

[18] Xu H., Cao W., Zhang X. (2013). Selenium-Containing Polymers: Promising Biomaterials for Controlled Release and Enzyme Mimics. Acc. Chem. Res..

[19] Yoshinaga N., Ishii T., Naito M., Endo T., Uchida S., Cabral H., Osada K., Kataoka K. (2017). Polyplex Micelles with Phenylboronate/Gluconamide Cross-Linking in the Core Exerting Promoted Gene Transfection through Spatiotemporal Responsivity to Intracellular pH and ATP Concentration. J. Am. Chem. Soc..

[20] Kanamala M., Wilson W.R., Yang M., Palmer B.D., Wu Z. (2016). Mechanisms and biomaterials in pH-responsive tumour targeted drug delivery: A review. Biomaterials.

[21] Zeng X., Liu G., Tao W., Ma Y., Zhang X., He F., Pan J., Mei L., Pan G. (2017). A Drug-Self-Gated Mesoporous Antitumor Nanoplatform Based on pH-Sensitive Dynamic Covalent Bond. Adv. Funct. Mater..

[22] Zou Y., Fang Y., Meng H., Meng F., Deng C., Zhang J., Zhong Z. (2016). Self-crosslinkable and intracellularly decrosslinkable biodegradable micellar nanoparticles: A robust, simple and multifunctional nanoplatform for high-efficiency targeted cancer chemotherapy. J. Control. Release.

[23] Zhai S., Hu X., Hu Y., Wu B., Xing D. (2017). Visible light-induced crosslinking and physiological stabilization of diselenide-rich nanoparticles for redox-responsive drug release and combination chemotherapy. Biomaterials.

[24] Ding Y., Du C., Qian J., Zhou L., Su Y., Zhang R., Dong C.-M. (2018). Tumor pH and intracellular reduction responsive polypeptide nanomedicine with a sheddable PEG corona and a disulfide-cross-linked core. Polym. Chem..

[25] Ma G., Liu J., He J., Zhang M., Ni P. (2018). Dual-Responsive Polyphosphoester-Doxorubicin Prodrug Containing a Diselenide Bond: Synthesis, Characterization, and Drug Delivery. ACS Biomater. Sci. Eng..

[26] Tian K., Jia X., Zhao X., Liu P. (2017). Biocompatible Reduction and pH Dual-Responsive Core Cross-Linked Micelles Based on Multifunctional Amphiphilic Linear–Hyperbranched Copolymer for Controlled Anticancer Drug Delivery. Mol. Pharm..

[27] Xiong D., Zhang R., Luo W., Gu H., Peng S., Zhang L. (2017). Hydrazone cross-linked micelles based on redox degradable block copolymer for enhanced stability and controlled drug release. React. Funct. Polym..

[28] Dai S., Ravi P., Tam K.C., Tam M.K. (2008). pH-Responsive polymers: Synthesis, properties and applications. Soft Matter.

[29] Luo Y.-L., Huang R.-J., Zhang L.-L., Xu F., Chen Y.-S. (2013). Dual-responsive polyacrylate copolymer micelles with PMAA and PNIPAAm graft brushes: Physicochemical properties and prednisone release. Colloids Surf. A Physicochem. Eng. Asp..

[30] Lin W., Yang C., Xue Z., Huang Y., Luo H., Zu X., Zhang L., Yi G., Yang C. (2018). Controlled construction of gold nanoparticles in situ from β-cyclodextrin based unimolecular micelles for in vitro computed tomography imaging. J. Colloid Interface Sci..

[31] Lin W., Zhang X., Qian L., Yao N., Pan Y., Zhang L. (2017). Doxorubicin-Loaded Unimolecular Micelle-Stabilized Gold Nanoparticles as a Theranostic Nanoplatform for Tumor-Targeted Chemotherapy and Computed Tomography Imaging. Biomacromolecules.

[32] Li Z., Day M., Ding J., Faid K. (2005). Synthesis and Characterization of Functional Methacrylate Copolymers and Their Application in Molecular Imprinting. Macromolecules.

[33] Yang Y.Q., Lin W.J., Zhao B., Wen X.F., Guo X.D., Zhang L.J. (2012). Synthesis and Physicochemical Characterization of Amphiphilic Triblock Copolymer Brush Containing pH-Sensitive Linkage for Oral Drug Delivery. Langmuir.

[34] Yang Y.Q., Guo X.D., Lin W.J., Zhang C.Y., Qian Y., Zhang L.J. (2012). Amphiphilic copolymer brush with random pH-sensitive/hydrophobic structure: Synthesis and self-assembled micelles for sustained drug delivery. Soft Matter.

[35] Wu L., Zou Y., Deng C., Cheng R., Meng F., Zhong Z. (2013). Intracellular release of doxorubicin from core-crosslinked polypeptide micelles triggered by both pH and reduction conditions. Biomaterials.

[36] Tian Y., Ravi P., Bromberg L., Hatton T.A., Tam K.C., Tam M.K. (2007). Synthesis and Aggregation Behavior of Pluronic F87/Poly(acrylic acid) Block Copolymer in the Presence of Doxorubicin. Langmuir.

[37] Zeng J., Du P., Liu L., Li J., Tian K., Jia X., Zhao X., Liu P. (2015). Superparamagnetic Reduction/pH/Temperature Multistimuli-Responsive Nanoparticles for Targeted and Controlled Antitumor Drug Delivery. Mol. Pharm..

[38] Tian K., Jia X., Zhao X., Liu P. (2016). pH/Reductant Dual-Responsive Core-Cross-Linked Micelles via Facile in Situ ATRP for Tumor-Targeted Delivery of Anticancer Drug with Enhanced Anticancer Efficiency. Mol. Pharm..

[39] Ritger P.L., Peppas N.A. (1987). A simple equation for description of solute release I. Fickian and non-fickian release from non-swellable devices in the form of slabs, spheres, cylinders or discs. J. Control. Release.

[40] Siepmann J., Peppas N. (2012). Modeling of drug release from delivery systems based on hydroxypropyl methylcellulose (HPMC). Adv. Drug Deliv. Rev..

[41] Lin W., Nie S., Zhong Q., Yang Y., Cai C., Wang J., Zhang L. (2014). Amphiphilic miktoarm star copolymer (PCL)3-(PDEAEMA-b-PPEGMA)3 as pH-sensitive micelles in the delivery of anticancer drug. J. Mater. Chem. B.

[42] Li Y., Li H., Wei M., Lu J., Jin L. (2009). pH-Responsive composite based on prednisone-block copolymer micelle intercalated inorganic layered matrix: Structure and in vitro drug release. Chem. Eng. J..

[43] Siepmann J., Peppas N.A. (2001). Mathematical modeling of controlled drug delivery. Adv. Drug Deliv. Rev..

[44] Ritger P.L., Peppas N.A. (1987). A simple equation for description of solute release II. Fickian and anomalous release from swellable devices. J. Control. Release.

[45] Ross P.D., Subramanian S. (1981). Thermodynamics of protein association reactions: Forces contributing to stability. Biochemistry.

